# Influence of Dentine Pre-Treatment by Sandblasting with Aluminum Oxide in Adhesive Restorations. An In Vitro Study

**DOI:** 10.3390/ma13133026

**Published:** 2020-07-07

**Authors:** Bruna Sinjari, Manlio Santilli, Gianmaria D’Addazio, Imena Rexhepi, Alessia Gigante, Sergio Caputi, Tonino Traini

**Affiliations:** 1 Department of Medical, Oral and Biotechnological Sciences, University “G. d’Annunzio” of Chieti-Pescara, 66100 Chieti, Italy; santilliman@gmail.com (M.S.); gianmariad@gmail.com (G.D.); imena.rexhepi@gmail.com (I.R.); allexia3191@gmail.com (A.G.); scaputi@unich.it (S.C.); t.traini@unich.it (T.T.); 2 Electron Microscopy Laboratory, University G. d’Annunzio Chieti-Pescara, 66100 Chieti, Italy

**Keywords:** adhesive dentistry, in vitro study, dentine pretreatment, fracture resistance, tensile stress test, sandblasting procedure

## Abstract

Dentine pretreatment through sandblasting procedures has been widely studied but no curve test results are currently available. Thus, the aim herein was to in vitro compare the adhesive strength in sandblasted or not samples using a universal testing machine. Thirty -two bovine teeth were divided into two groups, namely test (*n* = 16 bars), sandblasting with aluminum oxide particles (50 µm) was performed before the adhesion procedures), and control (*n* = 16 bars), where no sandblasting procedure was performed. A bi-material curve test was used to evaluate the characteristics of the dentine pretreatment in terms of tensile stress and fracture strength. A scanning electron microscope (SEM) was used to analyze the fracture topography in the composite, bonding, dentin, and at the relative interfaces. The results demonstrated a statistically significant difference between the two groups in terms of tensile stress at maximum load showing values of 84.300 ± 51.342 MPa and 35.071 ± 16.609 MPa, respectively for test and control groups (*p* = 0.033). Moreover, a fracture strength test showed values of 18.543 ± 8.145 MPa for test and 8.186 ± 2.833 MPa for control group (*p* = 0.008). In conclusion, the sandblasting treatment of the dentine significantly influenced the mechanical resistance of the adhesion in this in vitro study.

## 1. Introduction

The main purpose of dentistry, among others, is to restore patients’ aesthetics in compliance with function requirements and durability over the years [1,2]. The aesthetic improvement of tooth restoration is one of the main issues in clinical practice, and this has led to the research and development of dental materials and also clinical procedures able to best achieve this goal [3]. In the prosthetic and restorative dentistry fields, the use of adhesive materials has made it possible to increase the retention of the restorations, improve the aesthetic results, as well as reduce the invasiveness [4]. To date, the use of adhesive materials led to good outcomes both in terms of mechanical properties and adhesive capabilities [5]. However, several factors can influence the long-term performance of adhesive restorations, among which can be found the vulnerability of the adhesion interface to oral fluids, the adhesive technique used, the material and the quality of the restoration, and the treatment of the residual dental tissue [5,6]. In case of failure, the main effects involve the resin–dentin bonding and detachment of the restoration, as well as recurrent caries. The sum of these factors can reduce the survival rate of the bonded restorations [7]. However, adhesion is obtained through an exchange process in which minerals removed from the hard tissues are replaced by resin monomers, forming chemical bonds or micromechanical interlocking, called hybrid layer [8,9]. Comparing the bonding in enamel with that in dentin, we can note that it is particularly challenging due to the dentin composition, which has a lower inorganic component than enamel. Thus, the demineralized dentin collagen matrix plays a pivotal role for resin infiltration, forming the hybrid layer which allows bonding strength in the dentin [10]. Moreover, the disintegration of an adhesive interface over time is an important factor influencing the longevity of adhesive restorations [11]. The identification of defects at the tooth/restoration interface, represents an early sign of adhesive failure before the longevity of the restoration itself is compromised [12]. Over the years, different adhesion protocols, alternative materials, and substrate pretreatment procedures able to influence the degree of adhesion have been studied [13,14,15,16]. In this regard, chemical or mechanical dentin pretreatments are procedures that increase the roughness of the treated surface and may influence the adhesion strength of a bonding agent by increasing the contact area between the dentine and the adhesive surface [17]. In order to enhance the dentin bond strength during adhesive restorations, several studies have investigated various dentin surface pretreatment procedures. For example, related to chemical pretreatments, the application on the dentine surface of chlorhexidine after acid conditioning, seems to inhibit the activity of metalloproteinases [15]. On the other hand, based on the hypothesis that the elimination of the organic phase of the smear layer may improve the performance of the adhesive systems, the dentine pretreatment with sodium hypochlorite or hydrogen peroxide, have not obtained the desired results, especially when using self-etch adhesive systems [17]. 

The immediate dentin sealing (IDS) procedure was introduced with the aim of sealing the exposed dentinal tubules just after the tooth preparation to increase the adhesion strength through the bonding use [18]. Nevertheless, performing this technique, results in an interaction of the surface layer of the adhesive with the oxygen producing the oxygen-inhibition layer (OIL) [19]. Recently we reported a surface cleansing protocol capable of removing the OIL to prevent chemical interactions and adhesion with impression materials [20]. On the other hand, avoiding the use of the IDS technique can lead to an interaction of the dentine with the impression materials, producing tubules occlusion [21]. It has also been hypothesized to use air-polishing systems to cleanse the dental tissues, in order to increase their adhesiveness using materials such as sodium bicarbonate [22,23]. However, some authors argue that pretreatment with sodium bicarbonate causes, in contrast, a further accumulation of smear layer on the dentinal surface influencing negatively the adhesion of the restoration materials [22]. Whilst the glycine powder used for the pretreatment of dentine does not seem to cause significant weakening of the adhesive bond [24]. Among the above-mentioned dental pre-treatments that increase tooth cleansing is the sandblasting (air abrasion) procedure, which results in an increased roughness and adhesion [25,26]. The application of air abrasion with aluminum oxide increases the roughness of the dental surface which may be more conducive to bonding [27]. Several studies were carried out to evaluate the effectiveness of this treatment [25,26,27,28]. Motisuki et al. observed how air-abraded dentin, using 27-μm aluminum oxide particles, showed a higher bond strength than dentin prepared with the conventional method (bur in high-speed) [28]. Moreover, França et al. evaluated the bond strength, at different time points, of air-abraded dentine suggesting an improved result for pretreated samples [26]. 

Dentin bond strength is normally evaluated by conducting mechanical tests in tensile and/or shear mode at a certain level of crosshead speed [27,28]. The analysis of the quality of adhesion between composites, formed by a resin matrix with reinforcement elements, and an anisotropic substrate, such as that of the dentine, basically involves two aspects: (i) the determination of the adhesion strength and (ii) the fractography assessment related to the mechanical fracture itself. [29]. In fact, the non-homogeneous stress distribution between the components involved in the adhesion is a key factor in the development of the adhesion’s resistance [30,31]. In tensile tests, it is difficult to align the devices or the surfaces during the test performance to ensure a uniform distribution of the axial load. In addition, several cohesive dentin fractures were unfortunately observed during the preparation of the tensile tests [31]. Stress concentration is a primary aspect since the development of a fracture in a bi-material interface is characterized by microcracks nucleation and growth, determined by loading stress [31]. The performance of mechanical tests on the interface may provide only approximative measures since they are influenced - in the composite side - by the resistance capacity of the matrix with respect to the filler, while in the dentin, by pleiotropic factors such as the orientation of the collagen fibers, their degree of mineralization, and the distribution and direction of the dentinal tubules [30]. An alternative method for evaluating the mechanical characteristics of an adhesive system by fracture test is to use samples with a double curved notch as fracture trigger [32]. In this context, two fracture trigger zones are established on both composite and dentine sides. This sample design allows the fracture to occur normally with respect to the interface, thereby providing an important test to evaluate the adhesive strength.

To the authors best knowledge, there are no studies that analyze the sandblasting procedure through a curved test. Thus, the aim of this in vitro study was to compare adhesive strength in sandblasted or not sandblasted samples by means of a curved test in order to validate the correct clinical protocol of sandblasting procedure. 

The null hypothesis under test considered no differences in bond strength between the sandblasted and control samples.

## 2. Materials and Methods

### 2.1. Study Design

A total of 32 molars and premolars of the permanent bovine series, slaughter for human consumption, were used. Teeth selected showed absence of caries lesions or other infractions. The teeth samples obtained were randomly divided into 2 groups according to the following surface treatments:

- Group 1 (TEST): sandblasting with aluminum oxide particles (50 μm), etching treatment with 37% orthophosphoric acid (ENA Etch-Micerium), application of the adhesive (Prime & Bond—Etch & Rinse Adhesive-DENTSPLY) and a layer of composite (ENAMEL plus HRi-Micerium).
- Group 2 (CONTROL): etching with 37% orthophosphoric acid (ENA Etch-Micerium), application of the adhesive (Prime & Bond—Etch & Rinse Adhesive-DENTSPLY) and a layer of composite (ENAMEL plus HRi-Micerium).

### 2.2. Sample Preparation

**Figure 1:**
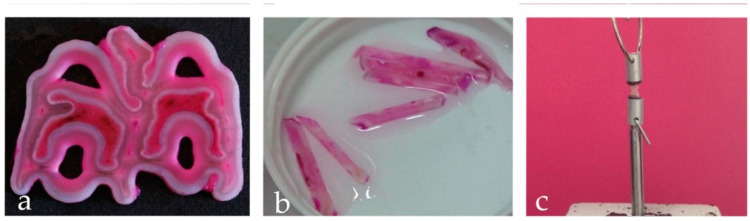
Sample preparation: (**a**) Detail of the crown’s cross section Shift reagent stained. Please note that this staining bonds the organic component highlighting the areas made up of dentin; (**b**) Dentine bars, size 2 × 2 × 8mm. (**c**) Sample mounted on the traction machine.

**Figure 2:**
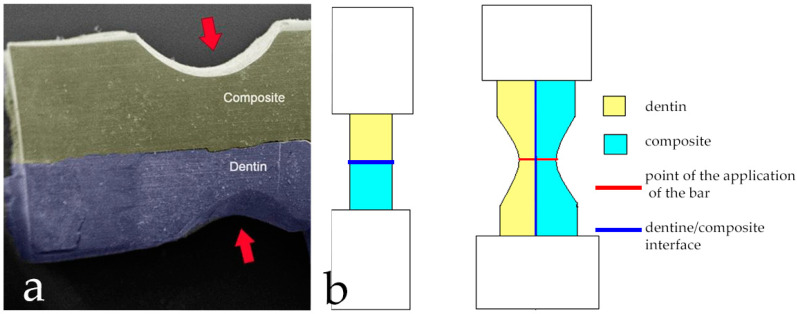
Notches creation: (**a**) The red arrows indicate the pre-crack areas; (**b**) Comparison between Conventional Test and bi-material Curved Test. In Curved Test, notches (created both on the dentine and composite sides) will be the pre-cracks from which the two fracture lines originate.

After the extraction, the teeth were stored in physiological solution at 4 °C. The crowns of the teeth were sectioned with a circular diamond blade microtome under water cooling (TT system TMA2, Grottammare, Italy). Then, the cuts were made by setting a blade rotation speed of 1500 rpm. Two mm thick cross sections were created. Subsequently, the crowns cross sections were subjected to staining with Shift reagent which, by binding to the organic component, highlighted the areas made up of dentin. After highlighting the letter, we proceeded to section them, using the same cutting method described above, creating 32 bars of dimensions 2 mm × 2 mm × 8 mm (Figure 1). All the samples obtained were cleaned in an ultrasonic bath (Digiclean, Tecnomed Italia S.r.l, Castelvecchio di Monte Porzio, Italy) with distilled water for 45 min. The bars were randomly divided into 2 groups of 16 pieces each. Each sample was equipped with an individual sample holder to standardize the extension treatment area (10 mm^2^) to be subsequently etched. On Test Group the pretreatment of the dentin surface was performed by sandblasting with aluminum dioxide particles. This procedure was carried out by placing the sandblaster tip at about 5 mm away from the surface of the sample to be treated, at an angle of about 90°. The sandblasting took place for about 10 s at a pressure of 0.2 MPa. After sandblasting, the acid etching was carried out by applying 37% orthophosphoric acid for about 20 s. Then, it was rinsed thoroughly with water for 30 s. The exposed surface was dried with an air jet for 3 s and the adhesive was applied with a microbrush. Finally, it was cured by a LED lamp (Valo, ultradent, Milano, Italy) at a power of 3.200 mW/cm^2^ for 3 s. Meanwhile, on the Control Group the dentin pretreatment was performed by acid etch with 37% orthophosphoric acid for 20 s, rinsing with water for 30 s and drying with air jet for 3 s. At this point, the adhesive was applied with a micro brush, which was then cured with a LED lamp (Valo, ultradent, Milano, Italy) at power of 3.200 mW/cm^2^ for 3 s. After carrying out these adhesive procedures, we proceeded for both groups with the application of a layer of composite resin (color UD3, ENAMEL plus HRi-Micerium). It was applied according to an incremental technique, which consists on the application of small quantities in order to avoid the formation of voids and allow a better polymerization. At each small quantity, the relative polymerization was carried out for 20 s using a LED lamp (Valo, ultradent, Milano, Italy) at a power of 1.000 mW/cm^2^. Once the thickness of 2 mm of composite was reached, the next layer was added till the completed reconstruction of the part. With a 3 mm diameter tungsten carbide bur, mounted on a contra-angle handpiece (E20C Expertmatic, Kavo Italia Srl, Genova, Italy) notches of about 2 mm were created at half of the bar length, both on the composite and dentine sides. These notches were performed to constitute crack triggers from which the two fracture lines would originate as shown in Figure 2. Each sample was subjected to polishing with a diamond paste of 25 micrometer particle size, with the aim to remove the surface irregularities. Subsequently, the specimens were let to an ultrasonic bath for 15 min and left immersed in physiological solution at 4 °C until to mechanical test was performed. 

### 2.3. Mechanical Test

A sample holder for the tensile test was prepared, creating anodized aluminum cylinders of 10 mm in length and 6 mm of external and 4 mm of internal diameters. At one end of the cylinder two parallel small holes of 1 mm in diameter were created. The two ends of each sample were inserted into a cylinder and fixed in this position using a dual resin composed of epoxy resin + bisphenol F epoxy resins mixed in a 1: 1 ratio with a hardener based on 3.6-diazotane-1.8- diamina (Bostik) as shown in Figure 2b. The tests were performed using a universal testing machine (LR30K; Lloyd Material Testing Bongor Regis, West Sussex, UK) set with Preload 1 N and Speed 0.5 mm/min. For each sample, the exact measurements of length and thickness were carried out with a digital caliper with a precision of ± 10 μm and have been registered in the appropriate window of the materials testing software (NEXYGENPlus, AMETEK (GB) Ltd., Bognor Regis, UK). The notches were oriented inside the specimen’s holder to remain in the middle of the samples to create a double fracture trigger. The mechanical test registered: the tensile stress at maximum load and stress at fracture. The collected results were expressed as means ± standard deviation (SD).

### 2.4. Scanning Electron Microscope (SEM) Test

Samples of both groups were examined under SEM (EVO 50 XVP with LaB6; Carl Zeiss S, Oberkochen, Germany). Samples were prepared and analyzed according to a previously described procedure [23,33]. Briefly, the samples were sputter coated with gold (K 550; Emitech Ltd., Ashford, Kent, UK) and mounted on a sample holder. The SEM equipped with a tetra solid-state back-scattered electron detector, was set to operate at 30 kV accelerating voltage, 10 mm working distance and 870 pA probe current. The images were captured with 20 scans using a line-average technique. These images were stored as TIFF files before their conversion using Image-Pro Plus version 6.0 (Media Cybernetics Inc., Bethesda, MD, USA) in order to obtain information about the mechanical characteristics of the fracture itself in the different layers: composite, bonding and dentin and at the corresponding interface.

### 2.5. Statistical Analysis

The data were statistically analyzed using a statistical software SPSS (V. 24-0-IBM Corp., Armonk, NY, USA). The mean and standard deviation values of all samples were calculated for the parameters of the maximum load stress tensile test and fracture stress tensile test. Descriptive statistics (Kolmogorov–Smirnov test) were used to evaluate whether they had a normal distribution. Student’s t-test for independent samples was performed. A *p*-value < 0.05 was considered statistically significant.

## 3. Results

**Figure 3:**
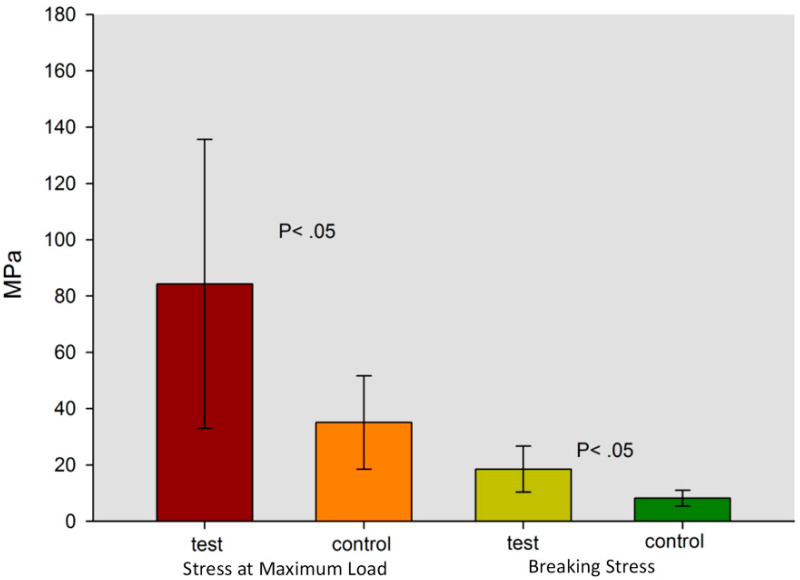
The graph shows the values of the two groups at Breaking Stress and at Maximum Load Stress.

**Figure 4:**
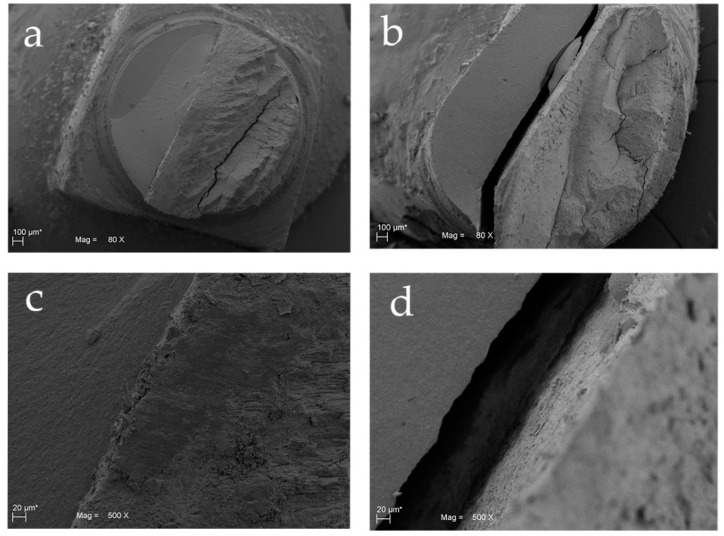
Scanning electron microscope (SEM) of Group 1 (Test) and Group 2 (Control): (**a**) SEM image of Group 1 at 80 × magnification. The fracture involved dentin with several direction changes; (**b**) SEM image of Group 2 at 80 × magnification. The fracture involved both dentin and the composite with direction changes; (**c**) Detail of Group 1 (interface) at 500 × magnification; (**d**) Detail of Group 2 at 500 × magnification.

**Figure 5:**
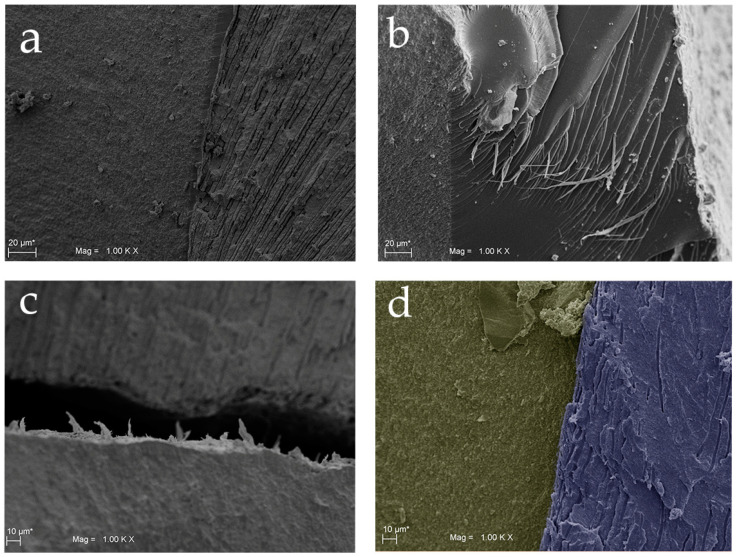
Scanning electron microscope images: (**a**) of Group 1 at 1.00K × magnification. At the interface there are no detachments; (**b**) SEM image of adhesive part, in detail observe the Hackle lines; (**c**) SEM image of Group 2 at 1.00K × magnification; it is shown a clear failure at the interface between dentin and adhesive; (**d**) 1.00K × magnification of both composite and dentin. sides.

Data obtained from tensile stress at maximum load tests were 84.300 ± 51.342 MPa for the Test Group and 35.071 ± 16.609 MPa for the control group. The statistical analysis, performed with *t*-test for independent samples, showed statistically significant differences between the two groups (*p* = 0.033) for this parameter. On the other hand, data obtained from the fracture stress test showed values of 18.543 ± 8.145 MPa for test group and 8.186 ± 2.833 MPa for control group. A statistically significant differences between the two groups (*p* = 0.008) was shown also in this test. These data are shown in Figure 3 where the mechanical characteristics of the fracture in the different layers, composite, adhesive, and dentin of both groups are indicated. Moreover, the results were confirmed also by SEM analysis, as shown in Figure 4 and Figure 5. Scanning electron microscopy analysis was performed at different magnifications, both for test and control samples. in Figure 4, it is possible to observe the surface microstructure of the test samples at different magnifications. It is clearly shown that no detachment occurred at the interface between the adhesive and the dentine. In addition, the SEM analysis demonstrated that the fracture involved the dentine was irregular, while, in the composite, the trend was more regular (Figure 4 and Figure 5). At the interface of test samples, there were no detachments shown and the contribution of the micro roughness induced by sandblasting appeared evident, as shown in Figure 4 and Figure 5. In particular, Figure 5b shows the layer of adhesive at 1.00 K magnification where the Hackle lines were evident, showing a sign of a fragile fracture in the layer of the adhesive itself, due to the low concentration of filling elements. Moreover, the SEM images of the Control group shown a clear failure at the interface between dentin and adhesive, thus recording a lower resistance to adhesion compared to the Test samples (Figure 4b,d and Figure 5c). On the other hand, Figure 4b showed how in the control samples the fracture involved both dentine and the composite parts. Figure 4d showed the sign of detachment at the interface at greater magnification.

## 4. Discussion

The null hypothesis under test was rejected. The results showed a statistically significant difference in terms of maximum load tensile stress and fracture tensile stress between the two groups. Moreover, the pre-treatment procedure of the dentinal surface produced a significant improvement in the adhesion resistance. In fact, the present study showed that the maximum load and the fracture tensile stress in the test group were higher compared to the control one. The present results are in accordance with Chaiyabutr et al. which evaluated the bond strength of self-adhesive luting cement used after dentine sandblasting compared to hand instrument excavation [34]. Similarly, Mujdeci et al. in another study showed how the shear bond strength to enamel and dentin significantly increased after airborne-particle abrasion treatment compared to the control groups [35]. Our SEM results demonstrated an evident increase of micro roughness induced by sandblasting procedure on test group as shown in Figure 4c and Figure 5a. These results are in accordance with Rafael et al. who found a greater roughness of the inter-tubular dentine after the dentin sandblasting procedure which leads to a greater adhesion contact surface. [36]. In addition to all these findings, D’Amario et al. assessed the effects of sandblasting on the micro tensile bond strengths of four commercial total-etch adhesives and demonstrated how this pretreatment procedure can increase the bond strength of the test groups [37]. Additional mechanical retention to the adhesive system appears to be related to the increased amount of the intertubular dentine available, thus expanding the contact area for adhesion. Moreover, Coli et al. stated that besides surface roughness, there are many other elements having effects on bonding, including the chemical composition of the dentin surface, orientation of the dentinal tubules and the formation of resin tags [17]. The present results are confirmed by several reported studies [34,35,36,37] using the curved test design. In the literature the concept of ‘average stress’ for measurements of the bond strength due to the non-uniform stresses exerted on the bonded interface was introduced [38]. Bond strength tests have been designed to transmit maximum stress in the shear apparatus along the interface, whereas the stresses for the tensile bond strength are propagated from the adhesive to the interface [38].

The force is transmitted through the adhesive, and partial cohesive failure, rather than interfacial failure, often occurs and the variation among samples might not validate the interfacial bond strength [39]. In this contest, the first difference between a traditional test and the tests used in this study results in the assembly of the specimen itself. Two important aspects of the tests’ design were the arrangement of the materials and the position of the interface between the two materials respect to the direction of the loading vectors. Using a traditional test, the interface between the materials usually is normal to the application of force and the two materials are placed above and over the interface [39]. Conversely, in a bi-material test, the specimen consists of materials placed side by side and the interface is parallel to the direction of the loading vectors, a necessary condition to best analyze the adhesion resistance.

Another pivotal aspect is given by the absence, in conventional tests, of defined pre-crack areas, which set a predetermined fracture trigger. This lack does not ensure that the fracture occurs exactly at the interface [40]. The tensile strength in conventional tests could be affected by parallelism and by the direction of the dentinal tubules compared to the interface [25,26]. Thus, the use of these tests may provide roughly measurements. A further aspect to be evaluated, which makes the curved test on bi-material specimens reliable to assess bonding effectiveness between composites and dentin, is given by the compound stress it generates due to shear and compression forces. In fact, these features allow to simulate the oral condition. Moreover, SEM analysis draw attention to the resistance properties of the different bi-material specimens which influenced the pattern of the fracture lines.

SEM analyses shown how in the composite side the fracture followed a slightly irregular trend given by the different range of matrix particles size. Once the fracture reached the adhesive layer, the propagation speed increases, and the failure pattern included the Hackle lines typical of glassy materials. When the fracture was observed in the Control specimens, a substantial failure between dentin and composite was marked while in the test samples there was no defined fracture between the two materials, but the fracture line followed a random pattern. In the dentin layer, on the other hand, the fracture has a much more irregular pattern than the composite side due to the pleiotropic factors linked to the morphology of the dentine itself, such as the orientation of the collagen fibers, their degree of mineralization and the distribution and direction of the dentinal tubules [41,42]. In addition, since the pre cracks areas were located at a bi-material interface, both tensile and shear stresses are induced around the crack tip and the crack will be stressed in two different modalities. In this way, we can determine the adhesive fracture energy and its directions. Furthermore, Franca et al. in their study demonstrated that air abrasion affects the fracture mode. Thus, the 46.67% of the fractures in the test group were cohesive in the adhesive side and this percentage was significantly higher than that of the other fracture modalities showing a reliable adhesion to the pre-treated dentin [26]. Cohesive failures in the adhesive side demonstrate an intact hybrid layer that protected the dentine. In contrast, adhesive failures highlighted a rupture at the dentin/resin bond interface, characterized by open dentinal tubules and demineralized intertubular dentin [43]. Moreover, Los and Barkmeier reported a high incidence of cohesive failures in aluminum oxide air treated surfaces after the shear bond strength test [44]. Therefore, our data demonstrated exactly what happens at the interface zone as, in the test specimens, there is no fracture line between the dentine and the adhesive and hence there was no detachment if not due to the random union of the fracture. The adhesion resistance to the interface was therefore significantly higher in the test samples. It is deducible that pretreatment by sandblasting with aluminum dioxide has increased and improved the performance in terms of adhesion. Although the novelty of this paper, the authors consider that it has different limitations. First of all, the authors did not used artificial aging, through critical thermal cycles to evaluate its effects on bond strength, which is an important variable to be considered. Moreover, further studies with an increased number of samples and an inclusion of other self-ecthing adhesives available on the market would be necessary to better understand the adhesion and to perform a comparative analysis between different bonding systems.

## 5. Conclusions

The results of this work show that the surface sandblasting treatment of the dentine before etching significantly influences the mechanical resistance of the adhesion to the interface. Thus, this procedure should probably be used in the adhesive cementations of restorations to the dentinal surface during the dental practice.
